# Supplementary material for: Effective Infection with Dengue Virus in Experimental Neonate and Adult Mice through the Intranasal Route
Source: Viruses. 2022 Jun 26;14(7):1394. doi: 10.3390/v14071394 (PMC9322762; doi:10.3390/v14071394)
Supplement: Supplementary file 1 [file viruses-14-01394-s001.zip › viruses-1733311-supplementary.pdf]

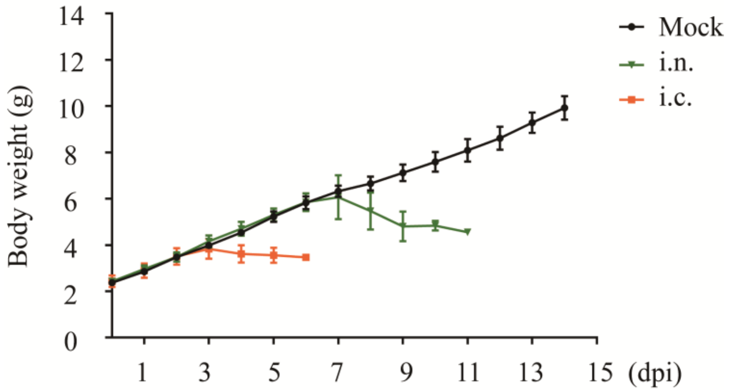

Figure S1. Body weight of Balb/c suckling mice post virus challenge. The data are expressed as mean ± SD from two independent experiments (titer=2.4×10^4 PFU, i.c., n=5; i.n., n=5; mock, n=5). Abbreviations: i.c., intracranial; i.n., intranasal; dpi, day post inoculation.

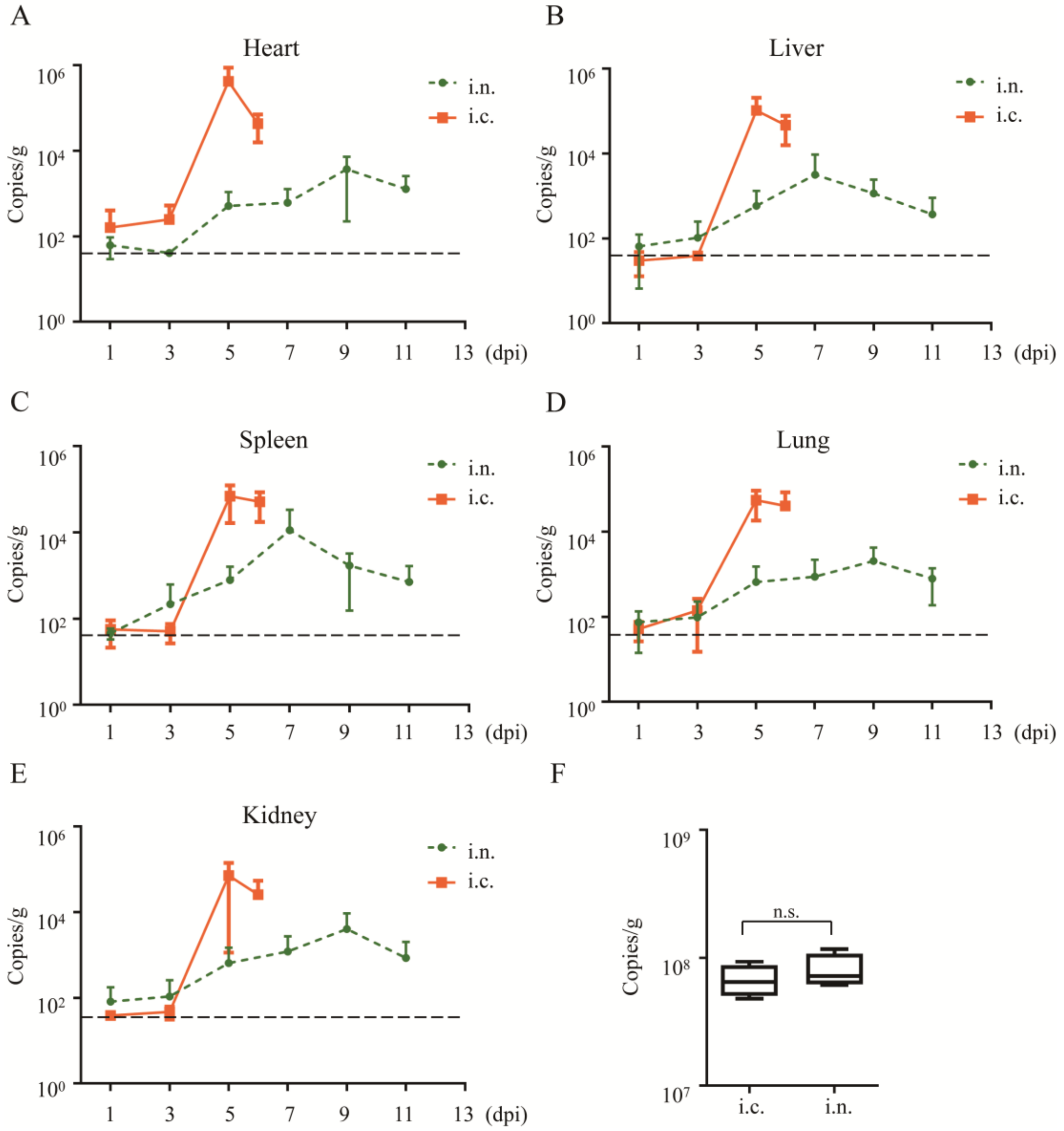

Figure S2. Viral RNAs loads in Balb/c suckling mice tissues. Heart (A), liver (B), spleen (C), lung (D) and kidney (E) were harvested and viral RNAs loads were determined by qRT-PCR (titer=2.4×10^4 PFU, i.c., n=5; i.n., n=5). B. Comparison of final RNA copies in mice brain between i.n. group and i.c. group. Data are expressed as mean ± SD. Abbreviations: i.c., intracranial; i.n., intranasal; dpi, day post inoculation.

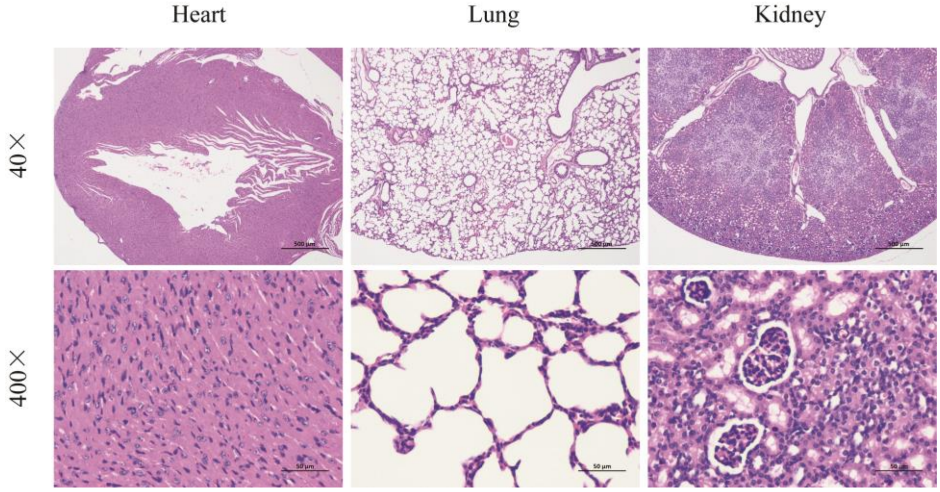

Figure S3. Histopathological study on heart, lung and kidney. No obvious histopathological changes were observed in these organs.

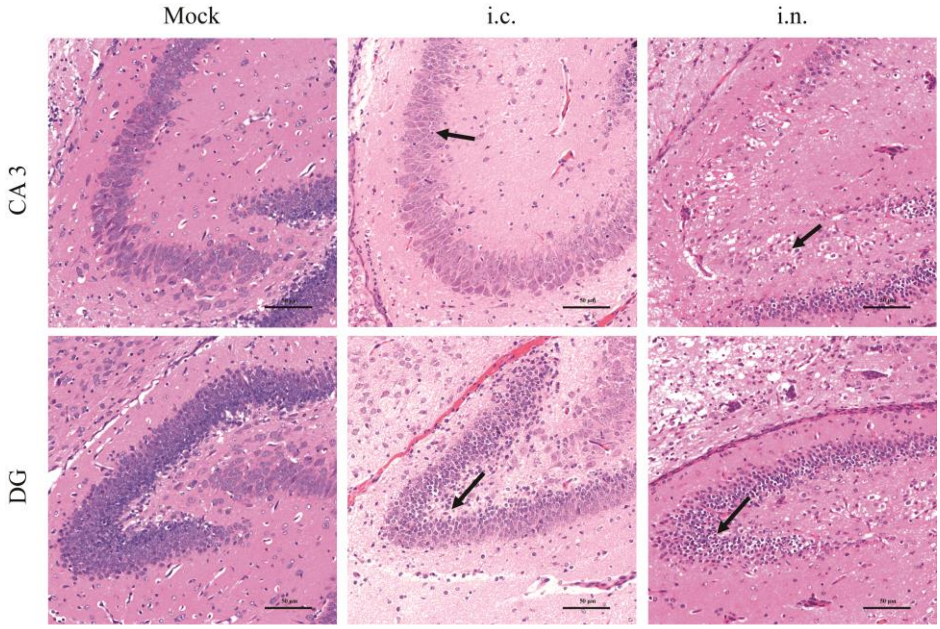

Figure S4. Histopathological changes in hippocampal CA3 and DG area. Brains were harvested from intracranially or intranasally challenged mice sacrificed at a late phase of illness (5 and 9 dpi, respectively) and stained with H&E. The histopathological changes on hippocampus CA3 and DG area

were observed (black arrows, necrotic pyramidal cells). Scal bar, 50 μm. Abbreviations: i.c., intracranial; i.n., intranasal.

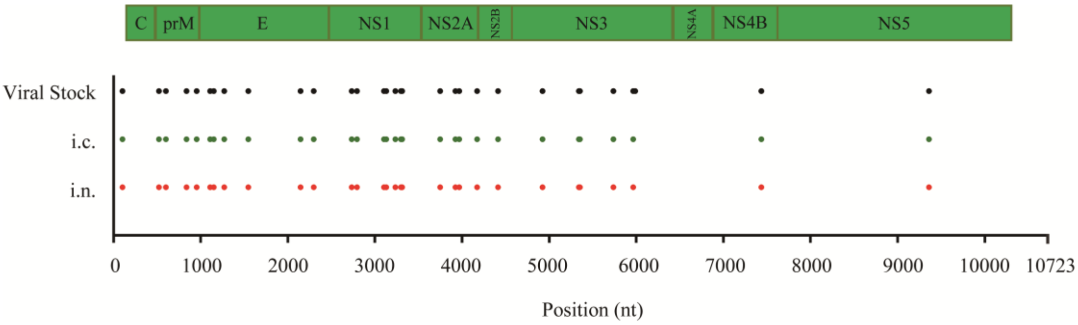

Figure S5. Gene Mutation of DENV-2 isolates. The genome sequences of DENV-2 isolates were analyzed by sequence alignment in the Genebank to identify the mutations. Mutation sites of each viral sample were represented as dots (black, viral stock; green, i.c.; red, i.n.). Abbreviations: i.c., intracranial; i.n., intranasal.

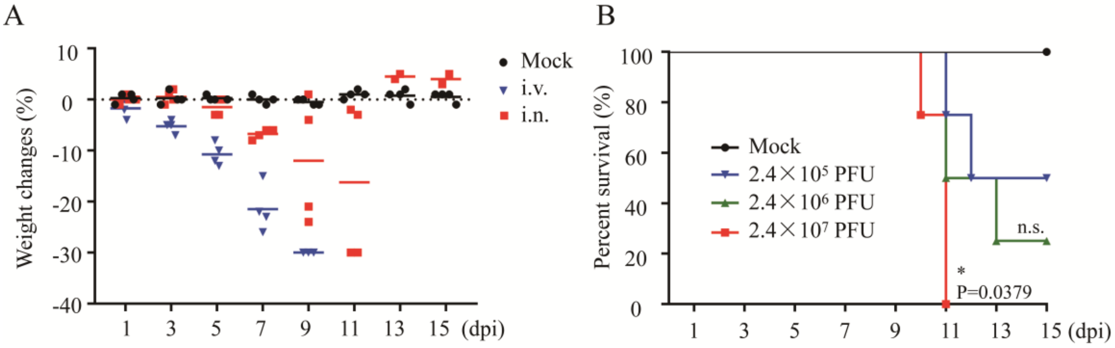

Figure S6. Clinical phenomenons of A6 mice after DENV-2 intranasal inoculation. (A) Body weight changes of A6 mice. (B) Survival probability of DENV 2 intranasally infected A6 mice with different titers (titer=2.4×10^5 PFU, n=4; titer=2.4×10^6 PFU, n=4; titer=2.4×10^7 PFU, n=4; mock, n=4). Abbreviations: i.v., intravenous; i.n., intranasal; dpi, day post inoculation. A log rank test was used to determine the statistical significance of survival compared with 2.4×10^5 PFU group, $P < 0.05$ indicates statistically significant differences.

Table S1. Clinical sign of intracranially or intranasally challenged suckling mice with DENV-2 (Titer = 2.4 × 10^4 PFU)

dpi	Clinical Sign, No./Total Observed					
	i.c.			i.n.		
	**Excitement**	**Asthenia**	**Death**	**Excitement**	**Asthenia**	**Death**
**4**	0/21	20/21	0/21	0/20	0/20	0/20
**5**	0/21	14/21	7/21	0/20	0/20	0/20
**6**	0/14	6/14	8/14	2/20	0/20	0/20
**7**	0/6	0/6	6/6	0/20	9/20	1/20
**8**				6/19	8/19	2/19
**9**				0/17	10/17	8/17
**10**				0/9	6/9	3/9
**11**				0/6	1/6	5/6
**12**				0/1	0/1	1/1

Abbreviations: i.c., intracranial; i.n., intranasal; dpi, day post inoculation.

Table S2. Cytopathic effects in Vero cells after DENV-2 inoculation

Cultured Sample		Day post inoculation						
		1	2	3	4	5	6	7
**Mock**	/	-	-	-	-	-	-	-
**i.c.**	Passage 1	-	-	+	++	+++	+++	++++
	Passage 2	-	-	-	-	+	++	+++
	Passage 3	-	-	-	+	++	+++	++++
**i.n.**	Passage 1	-	-	+	++	+++	+++	++++
	Passage 2	-	-	-	+	++	+++	++++
	Passage 3	-	-	+	++	+++	+++	++++

Abbreviations: i.c., intracranial; i.n., intranasal.

Table S3. Sequences of PCR primers used in this study

Primer name	Sequence (5'-3')
DENV-2-F	GCAGAATGCCCCAACACAAA
DENV-2-R	ACAAAATACATCCTGCCTTTCTCT
IL-1β-M-F	TGCCACCTTTTGACAGTGATG
IL-1β-M-R	TGATGTGCTGCTGCGAGATT
IL-6-M-F	ACCAGTGACTGAAAGACGCA
IL-6-M-R	TGGGGGAGGATGTTTGGATG
TNFα-M-F	ATGGCCTCCCTCTCATCAGT
TNFα-M-R	TTTGCTACGACGTGGGCTAC
CCL2-M-F	TTAAAAACCTGGATCGGAACCAA
CCL2-M-R	GCATTAGCTTCAGATTTACGGGT
CCL3-M-F	CTCCCAGCCAGGTGTCATTTT
CCL3-M-R	CAGGCATTCAGTTCCAGGTCAG
CCL4-M-F	GCTGCTTTGCCTACCTCTCC
CCL4-M-R	TCGAGTGACAAACACGACTGC
CCL5-M-F	TTTGCCTACCTCTCCCTCG
CCL5-M-R	CGACTGCAAGATTGGAGCACT
UBB-M-F	TTCGGTCTGCATTCCCAGTG
UBB-M-R	AACTTAAATTGGGGCAAGTGGC
